# Considerations in the management of hereditary angioedema due to C1-INH deficiency in women of childbearing age

**DOI:** 10.1186/s13223-022-00689-9

**Published:** 2022-07-13

**Authors:** Florence Ida Hsu, William Lumry, Marc Riedl, Raffi Tachdjian

**Affiliations:** 1 Yale University School of Medicine, New Haven, CT USA; 2 AARA Research Center, Dallas, TX USA; 3 University of California - San Diego, La Jolla, CA USA; 4 UCLA School of Medicine, Los Angeles, CA & Providence St. John Medical Center, Santa Monica, CA USA

## Abstract

Hereditary angioedema (HAE) is a rare, autosomal disorder that manifests with unpredictable episodes of severe swelling of the skin and mucous membranes. These attacks can be highly disfiguring and range in severity from mild to—in cases of airway swelling—life-threatening. Fluctuations in female sex hormones—such as the changes that occur during puberty, menses, contraceptive use, pregnancy, and menopause—can all affect the frequency and severity of HAE attacks. Disease management decisions for women of childbearing age may be more complex and require additional considerations since they could develop complications related to contraception, pregnancy, labor, delivery, and lactation. In addition, some HAE treatment options are contraindicated during pregnancy. Discussions about medications used to treat HAE should include a risk–benefit assessment of the woman’s health status, her preferences, and other factors that are relevant to the choice of therapy. Planning prophylactic therapies that are effective and safe before, during, and after pregnancy can prevent gaps in treatment, ensure continuity of care, and reduce both disease burden and risk of adverse fetal outcomes. The 2020 US Hereditary Angioedema Association (HAEA) Medical Advisory Board and 2021 World Allergy Organization/European Academy of Allergy and Immunology (WAO/EAACI) Guidelines outline key considerations for managing HAE in females of childbearing age (15–45 years), with the goal of improving treatment efficacy and safety for this cohort of patients. Treatment decisions made in a collaborative manner involving the patient, HAE specialist and obstetric/gynecologic specialist, is the best approach to ensure optimal HAE management and safety in this patient population.

## Introduction

Hereditary angioedema due to C1-INH deficiency (HAE-C1-INH) is a rare, autosomal disorder that manifests with unpredictable episodes of severe swelling of the skin and mucous membranes. These attacks can be highly disfiguring and range in severity from mild to—in cases of airway swelling—life-threatening. [1, 2]

HAE-C1-INH results from mutations in the *SERPING1* gene that affect C1 inhibitor production or function. Type 1 HAE-C1-INH is caused by decreased levels of C1 inhibitor (C1-INH), while Type 2 HAE-C1-INH stems from the presence of dysfunctional C1-INH. Low levels of functional C1-INH lead to altered regulation of the plasma contact system, which increases bradykinin production. Bradykinin is a vasoactive peptide that promotes increased vascular permeability, which manifests as swelling. [3]

Given HAE-C1-INH is an autosomal dominant hereditary disorder, both women and men should be equally affected. However, women generally present with more frequent and severe attacks of angioedema, compared with men [4–6]. Fluctuations in female sex hormones—such as the changes that occur during puberty, menses, contraceptive use, pregnancy, and menopause—can all affect the frequency and severity of HAE-C1-INH attacks. [7]

Compared to treatment of other patients with HAE-C1-INH, disease management decisions for women of childbearing age may be more complex and require additional considerations. Female patients may develop complications related to contraception, pregnancy, labor, delivery, and lactation. In addition, some HAE-C1-INH treatment options are contraindicated during pregnancy. [8]

Health care professionals should understand the implications that pregnancy may have on the clinical course of HAE-C1-INH. Women who are pregnant may experience more frequent and more severe abdominal attacks. The pain associated with an abdominal attack may be mistaken for pregnancy-related complications and lead to unnecessary diagnostic procedures. Disease control is critical and clinicians should pay particular attention to the efficacy and safety of on-demand and prophylactic therapies in these patients. Decisions about HAE-C1-INH treatment options must take into account the woman’s choices regarding family planning. This is especially important in women who are already on long-term prophylaxis (LTP), as some prophylactic therapies either lack safety data for use in pregnancy or are known to cause fetal harm. A birthing plan should be discussed with women who desire to become pregnant to ensure safe and comprehensive management of HAE-C1-INH throughout pregnancy.

In addition to these considerations, discussions about medications used to treat HAE-C1-INH should include a risk–benefit assessment of the woman’s health status, her preferences, and other factors that are relevant to the choice of LTP. Planning prophylactic therapies that are effective and safe before, during, and after pregnancy can prevent gaps in treatment, ensure continuity of care, and reduce both disease burden and risk of adverse fetal outcomes.

The 2020 US Hereditary Angioedema Association (HAEA) Medical Advisory Board Guidelines as well as the 2021 World Allergy Organization/European Academy of Allergy and Immunology (WAO/EAACI) Guidelines outline key considerations for managing HAE-C1-INH in females of childbearing age (15–45 years), with the goal of improving treatment efficacy and safety for this cohort of patients. [9, 10]

## Manifestations of HAE-C1-INH in women of childbearing age

Women with HAE-C1-INH experience attacks more frequently than men and their attacks also tend to be more severe [5–7]. In a study by Bork et al. (N = 209), significantly more women than men with HAE-C1-INH reported having ≥ 12 attacks per year (60.7% vs 43.6%; *P* < 0.02), and the mean number of attacks per year was higher in women (24) versus men (20.1) [5]. In a retrospective study (N = 193), Bouillet et al. found that women reported a higher percentage of their attacks to be severe (34.4% of attacks), compared with those reported by men (23.6% of attacks). [11]

Estrogen (exogenous and endogenous) can exacerbate the disease’s clinical manifestations. A review of case reports and clinical studies published between 2009 and 2019 concluded that estrogen is “an important precipitating and aggravating factor that triggers HAE-C1-INH attacks.^”^ [12] Hormonal fluctuations during puberty, menses, pregnancy, perimenopause, and menopause all affect the disease course and frequency of symptoms [7]. In a retrospective study of postpubertal women (N = 150) with HAE-C1-INH, most (62%) reported that their symptoms worsened during puberty; 35% reported that attacks were triggered by menstruation and 14% by ovulation [7]. In a study of factors that trigger attacks, women with HAE-C1-INH similarly identified menstruation, pregnancy, and ovulation as triggering factors [13]. Estrogen-containing oral contraceptives and hormone replacement therapy have also been identified as attack triggers in HAE-C1-INH, and initiation of estrogen therapy reportedly exacerbates symptoms in menopausal women [7, 8, 14, 15].

The impact of pregnancy on the frequency and severity of HAE-C1-INH attacks varies, even in the same woman with different pregnancies. Overall, more women report worsening of symptoms during pregnancy than report improvement or no change. Among 188 pregnancies in 41 women, when comparing disease severity with the 2 years prior to pregnancy, HAE-C1-INH symptoms worsened in 48%, were less severe in 33%, and remained unaffected in 19% [16]. In another study of 35 pregnancies in 22 women, attack rates increased in 29 of the 35 pregnancies, decreased in 4 pregnancies, and did not change in 1 pregnancy; there were no details for 1 pregnancy [17]. A third review of 125 pregnancies in 61 women found that, relative to baseline symptoms, attack frequency or duration of acute attacks increased in 59.2% of the pregnancies, improved in 14% of cases, and were unchanged in 26.4%. [18]

## Role of estrogens in HAE-C1-INH

Estrogen promotes HAE-C1-INH attacks by stimulating endothelial cells and augmenting activation of the prekallikrein–high-molecular-weight kininogen complex (prekallikrein-HMWK) [19]. Estrogen does this directly, stimulating the local release of heat-shock protein 90 (Hsp90) from endothelial cells; this action activates the prekallikrein-HMWK complex to form kallikrein. Kallikrein then cleaves the small peptide bradykinin from HMWK [19]. Bradykinin is a potent inducer of vasodilation and vascular permeability, and its interaction with venular B2 receptors is believed to be the final step in angioedema formation [19].

Estrogen may also play an important role in modulating bradykinin B2 receptor gene expression and function [20, 21]. In studies of female rats, estrogen regulated bradykinin B2 receptor levels in the rat uterus in both the myometrium and endometrium; B2 receptor levels were lowest when estrogen levels were low and highest when estrogen levels peaked [21].

## Diagnostic considerations

Individuals with HAE-C1-INH generally become symptomatic during adolescence or childhood, sometimes as early as 2 years of age. About 50% of patients manifest symptoms of swelling by age 10 years. The frequency and severity of attacks often increase after puberty, and almost all patients with HAE-C1-INH manifest symptoms by age 20 years. The mean age of the first attack is approximately 10 years [22].

In females, HAE-C1-INH symptoms may become more severe at puberty, as menstruation and ovulation can trigger abdominal attacks [5]. Symptoms of abdominal attacks in female adolescents—including abdominal pain, nausea, diarrhea, and vomiting—may mimic or overlap with symptoms of primary dysmenorrhea or other menses-related disorders [5, 23]. Diagnosis can thus be delayed when young women, or their health care providers, attribute HAE-C1-INH symptoms to menstrual-related pain. The opposite can also happen: after an HAE-C1-INH diagnosis, some abdominal symptoms/issues may be misattributed to HAE-C1-INH, leading to a delay in diagnosis of other gastrointestinal or gynecologic conditions.

In female adolescents, oral contraceptives may be prescribed to treat menstrual disorders such as menorrhagia, dysmenorrhea, or irregular periods. In some of these patients, HAE-C1-INH-associated swelling may occur for the first time with the introduction of estrogen-containing medications [22]. Angioedema related to estrogen-containing contraceptive use should prompt a workup for HAE-C1-INH.

## HAE-C1-INH treatment considerations in women of childbearing age

HAE-C1-INH management options include on-demand therapy to address acute attacks, short-term prophylaxis (STP) administered prior to medical or dental procedures, and LTP to prevent attacks [8]. Numerous treatment options are available in a variety of formulations. As with any treatment plan, health care providers should engage patients in a risk–benefit discussion about treatment options, patient status and preferences, and contraindications.

On-demand therapies for HAE-C1-INH include intravenous (IV) human plasma–derived (pd)C1-INH, IV recombinant human (rh)C1-INH, subcutaneous (SQ) icatibant, and SQ ecallantide (Table 1). Any of these therapies may be used as on-demand treatment in women of childbearing age who are not pregnant and do not have contraindications [9, 10]. Ecallantide must be administered by a health professional with access to appropriate medical support to manage potential anaphylactic reactions. Icatibant and the C1-INH therapies can be self-administered.

**Table 1:** On-demand and prophylaxis medications approved for HAE-C1-INH in the United States [9]

Brand name (Generic)	Class	Ages	Dosage	Route (self-administration)	Adverse effects
On-demand therapy					
Berinert (C1-INH) [46]	Plasma-derived C1-inhibitor	Pediatric and adults of all ages	20 U/kg	IV (yes)	Allergic reaction, nausea, diarrhea
Ruconest (C1-INH) [47]	Recombinant C1-inhibitor	Adolescents and adults	50 U/kg	IV (yes)	Headache, nausea, diarrhea
Kalbitor (ecallantide) [25]	Kallikrein inhibitor	≥ 12 years	30 mg	SQ (no)	Possible anaphylaxis (uncommon)
Firazyr (icatibant) [26]	B2 bradykinin receptor antagonist	≥ 18 years	30 mg	SQ (yes)	Redness, swelling, pain at the site of injection
Prophylaxis					
Danazol [39]	Attenuated androgen	≥ 18 years	Variable, with maximum long-term recommended dosage of 200 mg/day	Oral	Weight gain, virilization, hirsutism, acne, voice changes (hoarseness/deepening), menstrual irregularities, pseudomenopause, vaginal burning, vaginal dryness or itching, hypercholesterolemia, hypertension, hepatotoxicity
Cinryze (C1-INH) [48]	Plasma-derived C1-inhibitor	≥ 6 years	500 IU in children 6–11 years, 1000–2500 IU in adults, twice weekly	IV (yes)	Allergic reaction, nausea, diarrhea
Haegarda (C1-INH) [49]	Plasma-derived C1-inhibitor	≥ 6 years	60 IU/kg twice weekly	SQ (yes)	Injection site reactions
Takhzyro (lanadelumab-flyo) [41]	Plasma kallikrein inhibitor	≥ 12 years	300 mg every 2 weeks initially; may be given every 4 weeks based on clinical response	SQ (yes)	Injection site reactions, upper respiratory infections, headache
Orladeyo (berotralstat) [42]	Plasma kallikrein inhibitor	≥ 12 years	150 mg daily	Oral	Abdominal pain, vomiting, diarrhea, back pain, gastroesophageal reflux

Prophylactic options include IV or SQ C1-INH, SQ lanadelumab, and the recently approved oral agent berotralstat. Attenuated androgens such as danazol are recommended only as second-line HAE-C1-INH prophylactic agents due to potential for numerous significant adverse effects, and are contraindicated in pregnancy. C1-INH is the HAEA and WAO/EAACI guideline-preferred therapy for both on-demand and prophylactic treatment for pregnant and lactating women [9, 10].

When pregnancy is confirmed, the health care provider should initiate a discussion of the risks and benefits of LTP, including the potential for fetal exposure, especially in the first trimester, and the importance of effective HAE-C1-INH control during pregnancy [8, 24]. Re-evaluating the female patient’s current regimens, including their impact on continuity of care, may be necessary [24].

Many women with HAE-C1-INH – especially those with frequent or severe attacks – who become pregnant and are not on LTP may benefit from initiation of LTP during pregnancy [24]. Since pregnancy can have a variable impact on the clinical manifestations of HAE-C1-INH, the frequency of attacks during one pregnancy does not predict attack frequency during subsequent pregnancies [8]. The abdomen is the most commonly reported site for HAE-C1-INH attacks during pregnancy, possibly due to stretching of the uterus and fetal movement [9]. As abdominal attacks may be confused with other complications of pregnancy, their presence may be a consideration for starting prophylactic therapy during pregnancy in appropriate patients [8].

One additional consideration when discussing HAE-C1-INH management is the impact of LTP on quality of life. The HAEA and WAO/EAACI guidelines cite restoration of normal quality of life as a treatment goal [9, 10]. LTP is intended to reduce the burden of disease and the frequency and severity of HAE-C1-INH attacks. As HAE-C1-INH symptoms worsen in some women during pregnancy, these patients may derive particular benefit from prophylaxis [10].

Because HAE-C1-INH can affect many aspects of gynecologic care and vice versa, the woman’s HAE-C1-INH specialist should collaborate with her gynecologist or women’s health provider to ensure she receives the best possible care. When prescribing prophylactic treatment for women of childbearing age, consider the safety of a specific medication in early pregnancy and the potential effects of HAE-C1-INH therapies on fertility, pregnancy, and fetal development.

Data on the safety and effects of on-demand and prophylactic therapies during pregnancy, labor, delivery, and lactation generally are limited. With the exception of pdC1-INH, which has a long history of clinical use and safety, the available information comes primarily from case reports and anecdotal evidence.

### On-demand options

#### Ecallantide

Ecallantide, a kallikrein inhibitor, has not been studied in pregnant women. No published data are available for ecallantide use in pregnancy, labor, or delivery, and it is not known whether ecallantide is excreted in human milk [10, 25].

#### Icatibant

Icatibant, a B2 bradykinin receptor antagonist, has not been studied in pregnant women, and there are no data on the agent’s presence in human milk [26]. Anecdotal reports of icatibant use during pregnancy have been published. Hakl et al. reported that 6 women treated with icatibant during pregnancy delivered healthy babies (1 by caesarean section, 5 by spontaneous vaginal delivery) [27]. All pregnancies went to full term, and there were no complications during delivery. No congenital abnormalities were detected in the neonates [27]. Farkas et al. and Zanichelli et al. each reported a single case of a woman treated acutely with icatibant. One patient had Type 1 HAE-C1-INH, self-treated throughout her pregnancy, and delivered a healthy baby with no labor or delivery complications [22]. The other patient had Type 2 HAE-C1-INH, used icatibant to treat a laryngeal attack during her third pregnancy, and delivered a healthy baby [28].

#### C1-esterase inhibitor

In pregnant women, C1-INH is the preferred first-line therapy for on-demand treatment of HAE-C1-INH attacks and STP. The HAEA and the WAO/EAACI guidelines recommend C1-INH treatment for women who are pregnant [9, 10]. The recommendation for pdC1-INH is based on a long history of clinical experience, a well-established safety profile, and safety during pregnancy as documented by several case reports and observational studies in more than 120 women and more than 150 pregnancies [1, 7, 9, 17, 29, 30].

Use of rhC1-INH also may be considered for on-demand treatment of attacks in pregnancy. Though data for rhC1-INH are limited, the fact that rhC1-INH works by a similar mechanism as pdC1-INH is reassuring. Hakl et al. reported that pregnant women treated on demand with rhC1-INH (50 attacks) all delivered healthy, full-term babies; no complications during labor or delivery were reported [27]. Moldovan et al. also reported good efficacy and safety outcomes among pregnant women treated with rhC1-INH for acute attacks (N = 14) [31]. Patients for whom delivery methods were reported (n = 10, 8 vaginal, 2 cesarean) delivered full-term, healthy babies with no labor or delivery complications [31].

### Long-term prophylaxis options

#### Attenuated androgens

Attenuated androgens (AAs) such as danazol, stanozolol, oxandrolone, and methyltestosterone are highly effective as LTP. However, they can cause adverse effects that may be particularly problematic for women [32]. In addition to weight gain, dose-dependent side effects in women include hyperandrogenemia with possible virilization (clitoromegaly); hirsutism; hoarseness or deepening of the voice; menstrual irregularities; pseudomenopause; acne; burning, dryness, or itching of the vagina; and mood changes [33–38]. Androgens also may be associated with long-term adverse effects, including hypercholesterolemia, hypertension, and hepatotoxicity.

AAs are associated with fetal abnormalities and are contraindicated in pregnancy [39]. Women taking AAs as LTP should use additional contraception, regardless of whether they plan to conceive. Aside from their teratogenicity, AAs can impact a woman’s ability to conceive. Amenorrhea is a common adverse effect with AA use that can impact fertility. In a study of patients with HAE-C1-INH (N = 118) that included 58 women treated with danazol for 2 months to 30 years, amenorrhea was reported in 16 of 38 premenopausal women [32].

A negative pregnancy test result should be obtained before commencing AA therapy [39]. If a patient becomes pregnant while taking AAs, the drug should be discontinued and the patient and family informed about the risk of abnormalities of sexual differentiation in the fetus [24]. AAs are also contraindicated in lactating women [9].

Androgen use for managing HAE-C1-INH has declined with the availability of effective on-demand treatments and highly effective, more tolerable, long-term prophylactic therapies. [40].

#### Lanadelumab

Lanadelumab, a plasma kallikrein inhibitor, has not been studied in pregnant women and little is known about its effects on fertility, pregnancy, fetal development, or presence in human milk [41]. Monoclonal antibodies such as lanadelumab are transported across the placenta during the third trimester of pregnancy, so potential effects on a fetus are likely to be greater during this period [41].

#### Berotralstat

Berotralstat, the most recently approved option for HAE-C1-INH prophylaxis, is an oral plasma kallikrein inhibitor [42]. Berotralstat has not been studied in pregnant women, and there are no data on the presence of berotralstat in human milk [42].

## C1-Esterase Inhibitor (Plasma-Derived)

C1-INH is the HAEA and WAO/EAACI guideline–recommended prophylactic treatment for women who are pregnant [9, 10]. This recommendation is based on a long history of clinical use and literature reports of safe use during pregnancy.

PdC1-INH, administered IV or SQ, works by restoring C1-INH to levels that lower the risk of an attack [43]. Because pdC1-INH is a purified human protein designed to replace endogenous C1-INH and correct an underlying C1-INH deficiency or dysfunction, no adverse effects on fertility, pregnancy, or fetal development would be expected [24].

A 2020 review of IV pdC1-INH use during pregnancy for both on-demand and long-term prophylaxis reported outcomes in 91 women (136 pregnancies) who received a total of 1562 doses ranging from 500 to 3000 IU [30]. Infusions were administered during all 3 trimesters, but most commonly during the third trimester. Of the 128 fetuses for which outcomes were reported, 3 (2%) were lost to spontaneous abortion and 1 (1%) was stillborn [30]. Overall, pdC1-INH showed a favorable safety profile, with rates of congenital abnormalities similar to those of the general population [30].

Subcutaneous C1-INH prophylaxis was found to be safe and effective in women of childbearing age (n = 42) in the COMPACT open-label extension study [44]. Four women in that study were exposed to SQ C1-INH for up to 8 weeks during their first trimester, when the risk of teratogenic effects is greatest. All 4 patients delivered healthy babies with no congenital abnormalities [44]. The safety profile of SQ C1-INH for LTP during pregnancy is considered consistent with that of IV formulations, although studies to date have been limited [30].

SQ C1-INH administration is well-tolerated as routine prophylaxis, and has been found to improve health-related quality of life in people with HAE-C1-INH [45]. Compared to IV C1-INH prophylaxis, SQ C1-INH has substantially reduced the treatment burden and has made prophylaxis an attractive option for a larger segment of the HAE-C1-INH patient population, including women of childbearing age [45].

## Contraception in women with HAE-C1-INH

Consensus guidelines recommend that women with HAE-C1-INH avoid using contraceptives that contain estrogen. Use of estrogen-containing medications, including combined oral contraceptives (COCs), is associated with increases in factor XII, prekallikrein, kallikrein, and HMWK, any of which can increase bradykinin [32, 50–57]. Between 60 and 80% of women with HAE-C1-INH who use COCs experience more frequent and more severe attacks [8, 14, 58]. Similar effects can be expected with estrogen-containing patches and vaginal rings [6, 10].

Barrier methods, intrauterine devices (IUD), progestin-only pills (POPs), and progestins such as depot medroxyprogesterone acetate may be used [24]. POPs lead to symptom improvement in some women with HAE-C1-INH, are well-tolerated, and can be recommended for most patients with HAE-C1-INH [7, 8]. However, progestin doses used outside the United States are higher than those used in the US, so literature reports from abroad of androgenic effects and reduced HAE-C1-INH attacks with POPs may not apply in a US-based clinical setting [24, 59, 60].

## Conception planning in women with HAE-C1-INH

Patients with HAE-C1-INH may feel anxiety about passing the disease to their children, with some choosing not to have children or having fewer children than desired [61]. These patients should be reassured that highly effective therapies with reduced treatment burden are available, and that future therapies could make treatment even less burdensome. Genetic counseling should be offered to any patient of childbearing age with HAE-C1-INH [9, 24].

Conception planning should address the possibility of unplanned pregnancy and the female patient’s preferences with respect to contraception and conception. Questions to be considered include if/when the patient plans to become pregnant; how many children she wants; the type and efficacy of contraception she uses or plans to use; and whether fertility treatment is an option. In women with HAE-C1-INH who cannot conceive naturally, artificial insemination or in vitro fertilization (IVF) may increase the frequency and severity of attacks because of the increase in endogenous estrogens induced by injectable gonadotropins [24]. Current health status and treatment preferences should be part of a risk–benefit assessment when deciding on an HAE-C1-INH management plan and therapy.

Women with HAE-C1-INH who want to conceive and are using LTP may need to work with their health care provider to re-evaluate their current treatment plan. Women using C1-INH replacement therapy for LTP can continue treatment during conception, pregnancy, and delivery [9]. Attenuated androgens are contraindicated in women who are trying to conceive. Women taking lanadelumab for LTP should discontinue treatment before attempting conception [41]. Women using AAs or lanadelumab for LTP should be able to switch safely to C1-INH replacement therapy while attempting to conceive and continue C1-INH therapy during pregnancy, delivery, and lactation [9].

PdC1-INH is an appropriate on-demand treatment for women who are trying to conceive, including for those who require STP prior to artificial insemination or IVF. STP with pdC1-INH should be provided before IVF procedures that might trigger an attack, and C1-INH should be used on demand to treat an attack [24]. In a case report, prophylactic C1-INH was used successfully to prevent HAE-C1-INH attacks during IVF treatment and subsequently during pregnancy and delivery [62].

Prenatal diagnosis for C1-INH-HAE can be done if the disease causing mutation of the affected parent is known but this is rarely requested as the disease has a highly variable phenotype. Genetic testing can be performed using a chorionic villus or amniotic fluid sample taken after the 10th and 15th week of gestation, respectively. Chorionic villus sampling is preferred since testing can be done earlier in the course of pregnancy [24].

## HAE-C1-INH treatment considerations during pregnancy, labor/delivery, and postpartum

Women with HAE-C1-INH who become pregnant require vigilant care by an HAE-C1-INH expert in close cooperation with the patient’s obstetric/gynecologic health care professional. This patient-health care provider team should develop a plan for pregnancy, labor, and delivery, including:

- HAE-C1-INH management during pregnancy,
- Short-term prophylaxis for procedures during pregnancy, if needed,
- Method of delivery (eg, natural, cesarean section),
- Whether STP will be administered prior to delivery,
- Confirmation of access to on-demand C1-INH during labor and delivery,
- How an attack during labor or delivery will be managed,
- Postpartum management.

### HAE-C1-INH treatment considerations during pregnancy

As described earlier, HAE-C1-INH symptom frequency and severity may increase during pregnancy; attacks may also increase when the fetus has HAE-C1-INH, especially in the third trimester [24]. HAE-C1-INH attacks during pregnancy may be especially challenging for women with HAE-C1-INH. Abdominal attacks occur more frequently during pregnancy, and they often cause pain, nausea, vomiting, and diarrhea [5, 24]. These symptoms can compound pregnancy-associated gastrointestinal symptoms (“morning sickness”) and lead to dehydration. In addition, abdominal attacks that occur later in pregnancy may be mistaken for labor symptoms.

Health care providers should counsel women to contact their obstetrician if they are having abdominal pain and do not respond to HAE-C1-INH treatment, so that potential pregnancy complications can be quickly assessed [9]. Also, women need to be reassured that HAE-C1-INH does not increase the risk of a premature birth, spontaneous abortion, or cesarean delivery [7, 9, 18, 63].

### HAE-C1-INH treatment considerations during labor and delivery

Prior to delivery, the patient and her HAE-C1-INH provider, obstetrician, and anesthesiologist should discuss how to manage an attack. Preprocedural prophylaxis is generally unnecessary as attacks are uncommon during normal vaginal delivery, but medication should be available to the delivery room [7–9]. A preprocedural dose of pdC1-INH is recommended if the patient has had frequent symptoms in the third trimester; has a history of genital edema from mechanical trauma; or before forceps delivery, vacuum extraction, or cesarean section [9]. Endotracheal intubation should be avoided as this may produce laryngeal edema. If intubation is necessary, preprocedural prophylaxis is recommended [9, 24, 64].

Although the labor/delivery period is typically not associated with angioedema, HAE-C1-INH attacks may occur immediately after or within 48 h of delivery; some women may experience increased angioedema of the vulva. In these situations, pdC1-INH is the therapy of choice [9].

### HAE-C1-INH treatment considerations during lactation

Lactation may be associated with an increase in attacks, particularly abdominal attacks, which may be due to increased serum prolactin levels [16]. The HAEA and WAO/EAACI guidelines recommend C1-INH for on-demand treatment, STP, and LTP while the patient is breastfeeding (Table 2) [9, 10].

**Table 2:** US HAEA 2020 Guideline-Recommended Therapies for Women With HAE-C1-INH.^9^

	Not pregnant/not planning to be pregnant	Fertility treatment/planning pregnancy	Pregnancy	Labor/delivery	Breastfeeding
On demand	pdC1-INH, rhC1-INH, icatibant, ecallantide	pdC1-INH	pdC1-INH	pdC1-INH	pdC1-INH rhC1-INH
STP	pdC1-INH, anabolic androgens	pdC1-INH	pdC1-INH	pdC1-INH	pdC1-INH
LTP	pdC1-INH, lanadelumab	pdC1-INH	pdC1-INH	pdC1-INH	pdC1-INH

## Summary

The burden of HAE-C1-INH is higher in women, particularly those of childbearing age. Although several effective options for on-demand and prophylactic treatment exist, pdC1-INH therapy is recommended during pregnancy based on available long-term safety data. Because almost half of pregnancies are unplanned, it is important to discuss therapeutic options and their potential to affect fetal development, regardless of whether the patient is planning to conceive. Estrogen-containing contraceptives should be avoided in females with HAE-C1-INH.

While guidelines provide general treatment recommendations for women of childbearing age with HAE-C1-INH, they do not provide guidance on specific medications with respect to women who are planning to conceive, representing an unmet need for this patient population. However, the current guidelines do recommend C1-INH for women who are undergoing fertility treatments, who are pregnant, during labor and delivery, and during lactation. This allows for continuity of care before, during, and after pregnancy with no gaps in treatment, which is especially important as HAE-C1-INH attacks may increase at certain times during pregnancy and the postpartum period. Initiation of LTP is an individualized decision based on options, risks, and benefits. A collaborative approach that involves the patient, her HAE-C1-INH specialist, and her obstetric/gynecologic specialist is the best way to ensure optimal HAE-C1-INH management and safety in women of childbearing age.

## Data Availability

All data generated or analysed during this study are included in this published article (and its additional files).
