# Correction: Considerations in the management of hereditary angioedema due to C1-INH deficiency in women of childbearing age

**DOI:** 10.1186/s13223-022-00714-x

**Published:** 2022-09-03

**Authors:** Florence Ida Hsu, William Lumry, Marc Riedl, Raffi Tachdjian

**Affiliations:** 1 Yale University School of Medicine, New Haven, CT USA; 2 AARA Research Center, Dallas, TX USA; 3 University of California-San Diego, La Jolla, CA USA; 4 UCLA School of Medicine, Los Angeles, CA USA

## Correction: Allergy, Asthma & Clinical Immunology (2022) 18:64 https://doi.org/10.1186/s13223-022-00689-9

There is an error in the author affiliations listed in the final published version of this review. Only the first author is affiliated with Yale University School of Medicine. The other authors should have only one affiliation footnote listed for each (i.e., William Lumry^2^, Marc Riedl^3^ and Raffi Tachdjian^4^).

The original article has been corrected.
